# Conversion and Reversion Rates of Tuberculosis Screening Assays in Patients With Rheumatic Diseases and Negative Baseline Screening Under Long-Term Biologic Treatment

**DOI:** 10.20411/pai.v5i1.349

**Published:** 2020-02-26

**Authors:** Konstantinos Thomas, Emilia Hadziyannis, Chrisoula Hatzara, Anastasia Makris, Christina Tsalapaki, Argyro Lazarini, Kalliopi Klavdianou, Katerina Antonatou, Christos Koutsianas, Dimitrios Vassilopoulos

**Affiliations:** 1 Joint Rheumatology Program, Clinical Immunology-Rheumatology Unit; 2nd Department of Medicine and Laboratory; Hippokration General Hospital; National and Kapodistrian University of Athens School of Medicine; Athens, Greece

**Keywords:** Latent tuberculosis infection, rheumatic diseases, biologic DMARDs, tuberculin skin test, interferon-gamma releasing assays

## Abstract

**Background:**

To determine the conversion and reversion rates of tuberculosis (TB) screening tests (Tuberculin Skin Test-TST, Interferon Gamma Release Assay-IGRA: T-SPOT.TB) during biologic treatment in patients with rheumatic diseases and negative baseline screening.

**Methods:**

This was a long-term, longitudinal cohort study of 50 patients with rheumatic diseases and negative baseline TB screening (TST: < 5 mm, negative T-SPOT.TB) treated with tumor necrosis factor inhibitors (TNFi) or other non-TNFi biologics. Patients were rescreened at a mean time of 1.4 (first rescreening) and 6.9 (second rescreening) years from baseline, with both assays. The conversion (negative to positive) and reversion (positive to negative) rate was calculated for each TB screening test.

**Results:**

Fifty patients (mean age = 60 years) with various rheumatic diseases (rheumatoid arthritis: n = 24, spondyloarthropathies: n = 23, other: n = 3) were enrolled. During the first phase (baseline to first rescreening), all patients were treated with TNFi while during the second phase (first to second rescreening), TNFi (54%) and non-TNFi (46%) were used. Fifteen patients (30%) displayed conversion of at least 1 screening assay during follow-up (10 at the first and 5 at the second rescreening). This conversion rate was higher with TST (n = 11, 22% or 3.47/100 patient-years) compared to T-SPOT.TB (n = 4, 8% or 1.74/100 patient-years). Among the 10 converters at the first rescreening, 5 received isoniazid (INH) preventive therapy and 5 did not; an equal number of patients (3/5, 60%) reverted to negative with or without INH therapy. None of the patients developed active TB during follow-up (6.9 ± 1.0 years).

**Conclusions:**

Approximately one-third of patients with rheumatic diseases and negative baseline TB screening developed conversion of at least 1 screening test during long-term biologic treatment. This occurred most often with TST and was usually a transient event. These findings do not support routine serial TB retesting in biologic-treated patients with rheumatic diseases in the absence of TB risk factors.

## BACKGROUND

It is estimated that approximately one-quarter of the global population is infected by *Mycobacterium tuberculosis* [1], and although the lifetime risk of reactivation for an infected person is only 5%-10%, this reactivation of latent tuberculosis infection (LTBI) accounts for more than 80% of tuberculosis (TB) cases [2]. Prompt screening for LTBI has been the cornerstone for prevention of tuberculosis since the 1950s [3], and isoniazid (INH) preventive therapy has been the mainstay of treatment for more than 50 years, showing a 60%-90% reduction in TB cases [4].

Currently available assays for the diagnosis of LTBI include the tuberculin skin test (TST) and the interferon-gamma (IFN-γ) release assays (IGRAs) T-SPOT.TB (Oxford Immunotec, Oxford, UK) and QuantiFERON-TB Gold In Tube (QFT-GIT; Cellestis, Carnegie, Victoria, Australia). Compared to TST, IGRAs have shown similar sensitivity but higher specificity for the detection of LTBI, while both are negatively affected by immunosuppressive therapy [5]. Based on findings from several studies, recent guidelines recommend their use rather than TST as the diagnostic test of choice for individuals 5 years or older [6].

Tumor necrosis factor-α inhibitors (TNFi) were the first class of biologic disease-modifying anti-rheumatic drugs (bDMARDs) that were used in patients with rheumatic diseases, and they are currently licensed for different types of inflammatory diseases including rheumatoid arthritis (RA), spondyloarthropathies (SpA), inflammatory bowel disease (IBD) and psoriasis. The introduction of TNFi in clinical practice was followed initially by an increase in the TB cases in patients with rheumatic diseases undergoing this type of treatment [7-9], whereas cases of TB reactivation have been described less often in patients treated with non-TNFi bDMARDs [10] and the newer targeted synthetic DMARDs (tsDMARDs) [11], especially in high-incidence areas [12]. Following the initial reports of TNFi-induced TB reactivation, universal screening with TB screening tests of all patients with rheumatic diseases starting therapy with biologics has been employed and has proved to be efficacious in substantially decreasing the incidence of TB reactivation [13].

Despite these encouraging results, there are still a number of unresolved issues regarding TB screening in patients with rheumatic diseases. First, the optimal use of one or the other TB screening test has not been clarified. Both the World Health Organization (WHO) and the American College of Rheumatology (ACR) recommend screening with either TST or IGRA without preferentially advocating one technique over the other [14]. Experts in the field support the implementation of dual screening with both tests in patients with rheumatic diseases, because this approach has been found to increase sensitivity [15, 16], and it is also the practice for our unit [17]. Secondly, despite the recent ACR guidelines for annual rescreening of patients with RA treated with biologics and with negative baseline screening who have risk factors for TB exposure [14], the real-life data to support such a strategy are lacking.

Regardless of the rescreening approach employed, the rate of conversion and reversion of TB screening assays remains an issue. Recent data even with the IGRAs have shown that discordance in serial screening is not rare, especially in people with borderline positive results [18] as well as health care workers (HCWs) [19] and patients with rheumatic diseases [20-23].

Thus, the aim of our longitudinal cohort study was to evaluate the long-term rate of TB screening test conversion and reversion during biologic treatment. For this we utilized our previously published cohort of patients with rheumatic diseases and with negative screening at baseline [20].

## METHODS

### Patients

As previously reported, between October 2009 and December 2013, 247 patients with rheumatic diseases had been screened for LTBI before starting a TNFi in our unit of a tertiary referral hospital serving patients from across the country (Clinical Immunology-Rheumatology Unit, 2nd Department of Medicine and Laboratory, Hippokration General Hospital, Athens, Greece). Greece, according to the latest WHO data, is considered a low-TB-incidence country with an annual TB incidence rate of 4.5/100,000 [24]. Screening was performed with the TST, T-SPOT.TB, and QFTGIT [20]. All patients were HIV negative at baseline.

Seventy patients with negative baseline screening by all methods were reevaluated approximately 1 year later (first rescreening) while receiving TNFi treatment [20]. Among them, 20 were not available for a second rescreening: 2 had died, 5 denied rescreening, and 13 were lost to follow-up. Thus, 50 patients who were available for a second rescreening approximately 5 years later were included in the study.

During follow-up, patient demographics, history of new TB contact, history of *Bacillus Calmette– Guérin* (BCG) vaccination (based on the presence of BCG scar), and past and current immunosuppressive or disease-modifying therapy (glucocorticoids, conventional synthetic/csDMARDs and bDMARDs) were recorded. Physical examination, chest-computed tomography (CT) scan, or any other appropriate evaluation were performed in all patients who exhibited conversion of 1 TB screening test in order to rule out active TB. Patients who were considered as high risk for TB development after TST and/or T.SPOT-TB conversion were given LTBI therapy according to the decision of the treating physician.

All patients gave informed consent prior to rescreening, and the extension of the study was approved by the Hospitals' Institutional Review Board.

### TB screening tests (TST-IGRAs)

The TST was performed by intradermal injection (Mantoux method) of 0.1 mL (2 IU) of purified protein derivative (PPD RT 23; Statens Serum Institute, Copenhagen, Denmark) according to standard guidelines. A diameter of transverse induration ≥ 5 mm was considered positive. Blood for IGRAs was drawn the same day just before the TST. The T.SPOT-TB assay was performed and interpreted according to the manufacturer's instructions [20]. No invalid or indeterminate results were recorded in any of the tested samples. TST conversion was defined as the change of TST from < 5 mm to ≥ 5 mm while the opposite was regarded as reversion.

Since the beginning of the study, the QFT-GIT assay that was used for the baseline and first rescreening [20] has been replaced by the newer QuantiFERON-TB Gold Plus (QFT-Plus, Qiagen) assay which was used in the second rescreening. Since the TB antigens used in these 2 assays are not the same, the QFT results were not included in the current analysis. Nevertheless, we observed that 7 patients demonstrated a QFT conversion (14%, 4 at the first and 3 at the second rescreening). Among the 4 early converters, 1 received INH preventive therapy and remained positive while all 3 patients who did not receive INH therapy, reverted to negative at the second rescreening).

### Statistical Analysis

All statistical analyses were performed with SPSS (IBM SPSS Statistics for Windows, v. 20.0. Armonk, NY: IBM Corp). Continuous variables were expressed as mean ± SD or median (IQR) and dichotomous variables were expressed as absolute and percentage (%) values. Chi square or Fisher's exact test was used for comparison of dichotomous and Mann-Whitney or *t* test for continuous variables. Statistical significance was set at *P* < 0.05.

## RESULTS

### Patient Characteristics

**Figure 1.**
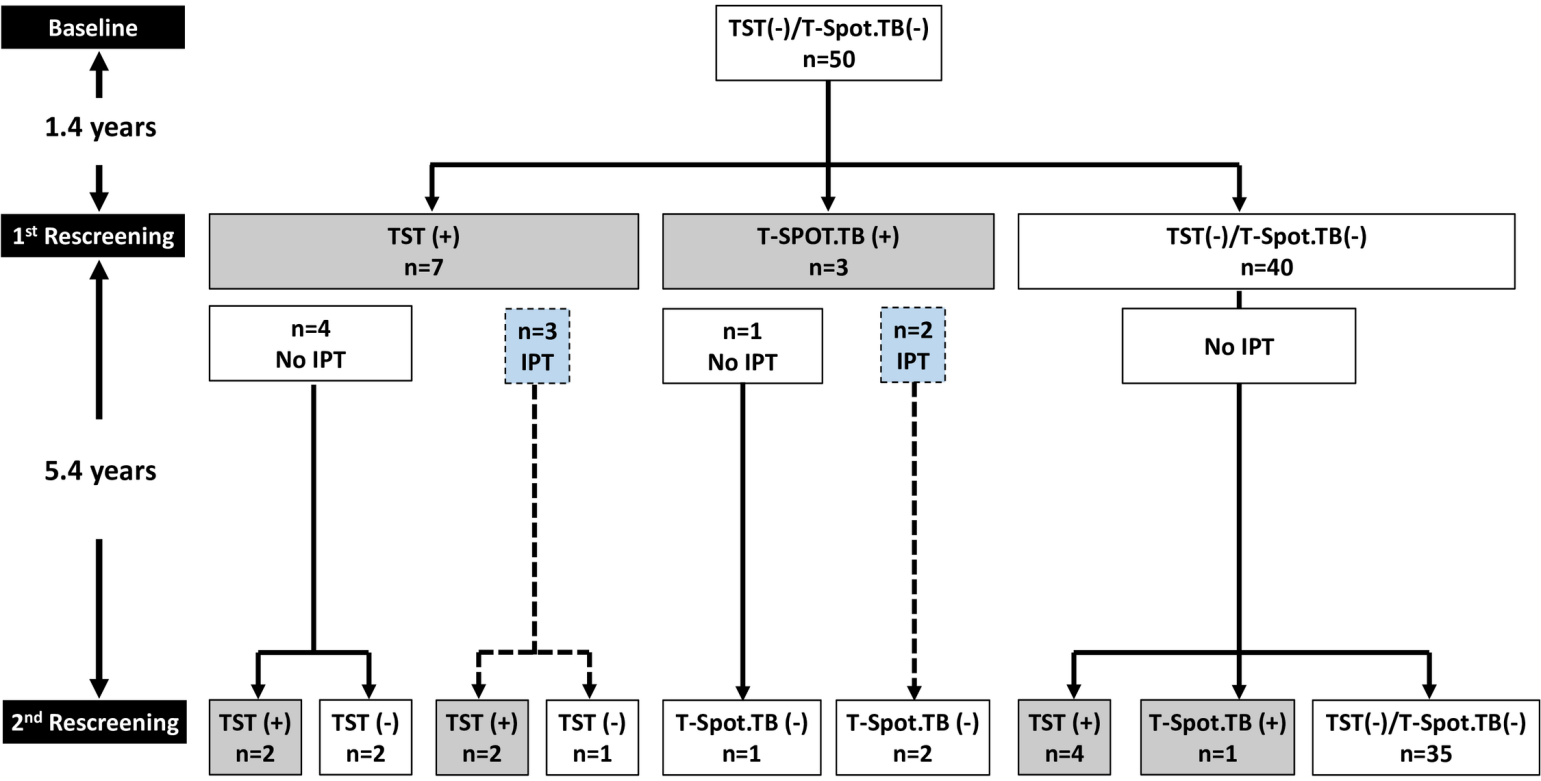
**Flow chart of rheumatic patients with negative baseline TB screening.** The flow chart of the 50 patients with negative baseline tuberculosis (TB) screening (by tuberculin skin test-TST and T-SPOT.TB) who were included in the study is depicted. The mean time (in years) between baseline and first rescreening as well as between the first and second rescreening is shown in the left side of the figure. Patients who converted at the first rescreening and were treated with IPT (isoniazid preventive therapy) are also shown (blue boxes and dashed lines).

Fifty patients with rheumatic diseases and negative baseline screening were included in the current study (Figure 1 and Table 1). All patients (n = 50) had their first rescreening 1.4 years (mean) and their second rescreening 6.9 years (mean) from baseline,. The characteristics of the 50 patients at the time of the second rescreening are shown in Table 1. Their mean age was 60 years, 31 (62%) were women, and their mean disease duration was 15 years. Rheumatoid arthritis (RA) was the most common diagnosis (n = 24, 48%) followed by spondyloarthopathies (SpA, n = 23, 46%) and other rheumatic diseases (n = 3, 6%).

**Table 1.** Patient characteristics.

Characteristic	n = 50
**Age,** years (mean ± 1 S.D.)	60 ± 13.6
**Female,** n (%)	31 (62%)
**History of BCG vaccination,** n (%)	15 (30%)
**Foreign nationality,** n (%)	5 (10%)
**Possible previous TB exposure**	2 (4%)
**Diagnosis,** n (%)	
RA	24 (48%)
AS	14 (28%)
PsA	9 (18%)
Other	3 (6%)
**Disease duration,** years (mean ± 1 S.D.)	15 ± 9.8
**Time interval between baseline - 1^st^ rescreening,** *years (mean ± 1 S.D.)*	1.4 ± 0.6
**Time interval between 1^st^ rescreening - 2^nd^ rescreening,** *years (mean ± 1 S.D.)*	5.4 ± 0.9
**Time interval between baseline and 2^nd^ rescreening,** *years (mean ± 1 S.D.)*	6.9 ± 1.0
**csDMARDs,** n (%)	19 (38%)
MTX	13 (26%)
LEF	5 (10%)
AZA	1 (2%)
**bDMARDs,** n (%)	47 (94%)
TNFi	27 (54%)
Non-TNFi	20 (46%)
RTX	6 (12%)
TCZ	6 (12%)
ABA	4 (8%)
SEC	2 (4%)
CAN	1 (2%)
UST	1 (2%)
**Type of treatment,** n (%)	
No treatment	1 (2%)
csDMARD monotherapy	2 (4%)
csDMARD + TNFi	7 (14%)
TNFi monotherapy	20 (40%)
csDMARD + non-TNFi	10 (20%)
non-TNFi monotherapy	10 (20%)
**Glucocorticoids,** n (%)	8 (16%)
**Mean daily dose,** (mean ± 1 S.D.)	5 ± 1.6 mg

The characteristics of the 50 patients who were included in the study are shown. BCG: Bacillus Calmette–Guérin; RA: rheumatoid arthritis; AS: ankylosing spondylitis; PsA: psoriatic arthritis; csDMARDs: conventional synthetic disease modifying drugs; bDMARDs: biologic disease modifying drugs; TNFi: tumor necrosis factor inhibitor; RTX: rituximab; TCZ: tocilizumab; ABA: abatacept; SEC: secukinumab; CAN: canakinumab; UST: ustekinumab.

All patients received TNFi during the first phase (baseline to first rescreening) as previously described [20] while during the second phase (first to second rescreening) almost all (47/50, 94%) were treated with bDMARDs (TNFi: 54%, non-TNFi: 46%), 2 (4%) received only csDMARDs, and 1 (2%) did not receive any additional therapy. At the time of the second rescreening, only 8 patients (16%) were receiving glucocorticoids with a mean prednisolone equivalent daily dose of 5 mg.

Regarding TB risk factors, 5 patients (10%) were from countries with high TB incidence while only 2 (4%) reported possible TB exposure during follow-up. Fifteen (30%) patients had been previously vaccinated with BCG (usually during their childhood or early adolescent period).

### TB Screening Test Conversion and Reversion During Follow-Up

During the follow-up period (~ 6.9 years), 15 (30%) patients with negative baseline TB screening, showed conversion in at least 1 TB screening test (Figure 1). Their detailed characteristics are shown in Table 2.

**Table 2.** Characteristics of patients that converted at least 1 TB screening test (n=15).

#	Sex/Age	Diagnosis	TB Risk Factors	BCG	TNFi Between Baseline and 1^st^ Rescreening	1^st^ rescreening			bDMARD Between 1st and 2nd Rescreening	2nd Rescreening			Type of Conversion
						Gluco-Corticoids*	TST Diameter	T-SPOT.TB		TST Diameter	T-SPOT.TB	bDMARD	
**1**	**F/79**	**RA**	**Yes**	**No**	**TNFi** (ADA)	**Yes (5 mg/d)**	**15**	**Negative**	**RTX**	**8**	**Negative**	**-**	**Persistent converter**
**2**	**M/49**	**PsA**	**No**	**No**	**TNFi** (ETN)	**No**	**10**	**Negative**	**TNFi** (ETN)	**18**	**Negative**	**TNFi** (ETN)	**Persistent converter**
**3**	**F/79**	**RA**	**No**	**No**	**TNFi** (CZP)	**Yes (5 mg/d)**	**0**	**8 spots (panel A)**	**RTX**	**0**	**Negative**	**RTX**	**Transient converter**
**4**	**M/65**	**PsA**	**No**	**No**	**TNFi** (ETN)	**No**	**10**	**Negative**	**TNFi** (ETN)	**0**	**Negative**	**TNFi** (ETN)	**Transient converter**
**5**	**F/80**	**RA**	**No**	**No**	**TNFi** (GOL)	**Yes (5 mg/d)**	**10**	**Negative**	**TNFi** (GOL, CZP, ETN) **RTX** **TCZ**	**0**	**Negative**	**TCZ**	**Transient converter**
**6**	**M/48**	**AS**	**No**	**Yes**	**TNFi** (INF)	**No**	**0**	**Negative**	**TNFi** (INF)	**12**	**Negative**	**TNFi** (INF)	**Late converter**
**7**	**M/62**	**AS**	**No**	**Yes**	**TNFi** (INF)	**No**	**0**	**Negative**	**TNFi** (ADA)	**5**	**Negative**	**TNFi** (ADA)	**Late converter**
**8**	**F/64**	**PsA**	**No**	**No**	**TNFi** (INF)	**No**	**0**	**Negative**	**SEC**	**7**	**Negative**	**SEC**	**Late converter**
**9**	**F/59**	**PsA**	**No**	**No**	**TNFi** **(ADA)**	**Yes (5 mg/d)**	**0**	**Negative**	**TNFi** (GOL, ETN) **UST**	**6**	**Negative**	**SEC**	**Late converter**
**10**	**M/57**	**PG**	**No**	**No**	**TNFi** (INF)	**Yes (5 mg/d)**	**0**	**Negative**	**TNFi** (INF)	**0**	**>50 spots Panels A and B**	**TNFi** (INF)	**Late converter**
**11**	**F/56**	**AS**	**No**	**No**	**TNFi** (ADA)	**No**	**0**	**8 spots (panel A)**	**TNFi** (INF)	**0**	**Negative**	**TNFi** (INF)	**Early converter -INH therapy**
**12**	**F/78**	**RA**	**Yes**	**Yes**	**TNFi** (ADA)	**No**	**0**	**15 spots (panel B)**	**TNFi** (ADA)	**0**	**Negative**	**TNFi** (ADA)	**Early converter -INH therapy**
**13**	**F/66**	**RA**	**No**	**Yes**	**TNFi** (ADA)	**No**	**13**	**Negative**	**TNFi** (CZP)	**18**	**Negative**	**TNFi** (CZP)	**Early converter -INH therapy**
**14**	**M/37**	**AOSD**	**No**	**Yes**	**TNFi** (ETN)	**Yes (5 mg/d)**	**15**	**Negative**	**TNFi** (ETN) **ANA** **TCZ**	**15**	**Negative**	**CAN**	**Early converter -INH therapy**
**15**	**F/53**	**AS**	**No**	**No**	**TNFi** (INF)	**No**	**11**	**Negative**	**TNFi** (INF)	**0**	**Negative**	**TNFi** (INF)	**Early converter -INH therapy**

The characteristics of the 15 patients who converted TST or T-SPOT.TB (grey boxes) are shown. This includes those who remained positive at both re-screenings (“persistent converters”, n=2, #1-2)), those who reversed from positive to negative between the first and second rescreening without isoniazid (INH) therapy (“transient converters”, n=3, #3-5), those who converted between the first and second rescreening (“late converters”, n=5, #6-10), and finally the converters at the first rescreening who received INH therapy (“early converters on INH therapy”, n=5, #11-15). * Expressed as prednisolone equivalent dose (in mg/day) F: female; M: male; TB: tuberculosis; BCG: Bacillus Calmette–Guérin; TST: tuberculin skin test; csDMARDs: conventional synthetic disease modifying drugs; bDMARDs: biologic disease modifying drugs; INH: isoniazid; RA: rheumatoid arthritis; AS: ankylosing spondylitis; PsA: psoriatic arthritis; AOSD: adult-onset Still's disease; LEFL: leflunomide; MTX: methotrexate; AZA: azathioprine; INF: infliximab; ADA: adalimumab; RTX: rituximab; ETN: etanercept; TCZ: tocilizumab; SEC: secukinumab; CZP: certolizumab pegol; CAN: canakinumab; GOL: golimumab; ANA: anakinra

Ten patients (20%) exhibited conversion at their first rescreening at 1.4 years (TST: n = 7, T-SPOT. TB: n = 3) and 5 (10%) at their second rescreening at 6.9 years (TST: n = 4, T-SPOT.TB: n = 1, late converters). The median TST diameter of converted patients was 10.5 mm (IQR: 9.25-13.5). Among the 10 converters at the first rescreening, 5 received INH preventive therapy and 5 did not, according to the decision of their physicians. Among the 5 converters at the first rescreening who did not receive INH, 3 (60%) reverted to negative (transient converters, TST n = 2, T.SPOT-TB n = 1) at the second rescreening while an equal number of patients who received INH preventive therapy reverted to negative (n = 3, 60%, TST n = 1, T.SPOT-TB n = 2, early converters – INH therapy, Figure 1 and Table 2).

Thus, only 2 (33%) patients who had a conversion at first rescreening remained positive at the end of follow-up (both with TST, persistent converters). None of these 50 patients developed active TB during the follow-up period.

### Comparison Between Converters and Non-Converters

There were no statistically significant differences between converters (n = 15) and non-converters (n = 35) as shown in Table 3, although there was a trend for increasing age (62.2 ± 12.9 vs 57.9 ± 13.9 years) and a non-RA diagnosis (67% vs 46%) for converters vs non-converters, respectively.

**Table 3.** Comparison between converters and non-converters.

Characteristic	Non-Converters n = 35	Converters n = 15	*P*
**Age (years), mean ± SD**	57.9 ± 13.9	62.2 ± 12.9	0.27
**Female, n (%)**	22 (63%)	9 (60%)	1.0
**History of BCG vaccination, n (%)**	10 (28.6%)	5 (33.3%)	0.74
**Foreign nationality**	2 (5.7%)	3 (20%)	0.15
**Possible exposure**	1 (2.9%)	1 (6.7%)	0.51
**Diagnosis**			
**RA**	19 (54%)	5 (33%)	
**Non-RA**	16 (46%)	10 (67%)	0.22
**AS**	10 (28.6%)	4 (26.7%)	
**PsA**	5 (14.3%)	4 (26.7%)	
**Other**	1 (2.9%)	2 (13.3%)	
**Disease duration (years), mean ± SD**	14.3 ± 7.8	16.7 ± 13.6	0.44

The comparison between those who did (n = 15) or did not (n = 35) convert during follow-up is shown.

BCG: Bacillus Calmette–Guérin; RA: rheumatoid arthritis; AS: ankylosing spondylitis; PsA: psoriatic arthritis.

### TST and T-SPOT.TB Conversion Rates

Overall, more patients displayed a TST (n = 11, 22%) compared to T-SPOT.TB conversion (n = 4 or 8%), although this did not achieve statistical significance *(P* = 0.09). The rate of conversion decreased over time for both tests, although this was higher for TST (14%, 7/50 between baseline and first rescreening vs 10%, 4/40 between first and second rescreening) than T-SPOT.TB (6%, 3/50 vs 2.5%, 1/40, respectively).

Similarly, the conversion rate per 100 patient-years from baseline to first rescreening (70.9 patient-years) was 9.87 for TST and 5.64 for T-SPOT.TB, whereas the rates for the total duration of follow-up (288 patient-years) were 3.47 and 1.74 per 100 patient-years, respectively (OR = 2, 95% CI: 0.68-6.47, *P* = 0.3).

## DISCUSSION

To our knowledge, this is the first prospective, clinical practice based, long-term (~7 years) study that evaluated the conversion and reversion rates of 2 different TB screening assays (TST and IGRA/T-SPOT.TB) during serial retesting in biologic-treated patients with rheumatic diseases and negative baseline screening. Our main findings are that in a low risk population, conversion of a TB assay during biologic treatment is common (occurring in 30% of patients), and it is more frequent with TST than an IGRA and is usually a transient event.

TB screening with TST and/or IGRAs is currently recommended as a standard procedure for all patients with rheumatic diseases starting biologic or targeted synthetic (such as kinase inhibitors) therapy [14], because of the high incidence of TB reactivation that was noticed in the early years of TNFi therapies without appropriate TB screening for LTBI [7]. Nevertheless, in real life settings it has been shown that even among patients with negative baseline screening, TB can occur during biologic treatment. Its incidence varies significantly based mainly on the TB incidence in the respective population (0.2%-4%) [12, 25, 26]. In a recent study from a highly endemic area (South Africa), the rate of active TB was 1.2/100 patient-years among biologic-users; half of the active TB cases were due to new TB exposure and half were due to TB reactivation (indicative of TB screening failure) [12].

Although TB screening is now a routine procedure for all patients starting biologics, the value of routine serial retesting during long-term biologic therapy has not been studied adequately. Some scientific societies recommend rescreening only for high-risk patients [14, 27, 28] whereas others do not offer any specific recommendations [27]. Despite these recommendations though, in daily clinical practice the majority of rheumatologists (63%) in a recent survey, reported serial TB retesting of biologic-treated patients with rheumatic diseases (24% annually for all patients, 15% every 2 years and 24% only for those with TB risk factors) [29]. Interestingly, this practice was more common in the United States (70%) than outside the United States (31%) [29].

There are several unresolved issues regarding serial retesting in biologic-treated patients with negative baseline screening. The first issue is the conversion rate with the different TB screening tests. Data from the literature have shown that the conversion rate is higher for TST (0-37%) [20, 30-38] than the T-SPOT.TB (0-15%)[20, 36, 39] and the QFT-GIT (0-12%) [20-23, 33-36, 40, 41] tests. Similar findings were observed in our cohort study where during the 7- year follow-up period, 30% of patients converted in at least 1 TB screening assay. The rate was higher for TST (22%) than T-SPOT.TB (8%). Interestingly, this conversion rate appeared to decrease gradually for both assays over the years.

It should be noted that this high conversion rate occurred in a low-TB-risk patient population (only 2/50 patients had potential TB exposure during the follow-up period) residing in a low-TB-incidence country [24].

Data from different countries have shown that the conversion rate of IGRAs may not differ significantly between countries with low or high TB incidence [20, 21, 23, 26, 42] but as Kim *et al* recently reported, in high-incidence countries the rate of active TB development among converters is higher [41].

The potential practical implications of our findings are that the demonstration of a TB test conversion during serial retesting of biologic-treated patients with rheumatic diseases will require extensive evaluation (including sputum stains and cultures, CT of the chest, additional lab work etc.) for active TB of a large proportion of them. Furthermore, patients with a negative workup for active TB should receive appropriate preventive therapy (INH or rifampin) for 6 to 9 months with close clinical and laboratory monitoring, which would increase cost and the possibility of drug-related adverse events.

A second unresolved issue with serial TB retesting is whether these represent true or transient conversions. Most data so far are derived from serial retesting in otherwise healthy HCWs. These data have shown a high reversion rate (form positive to negative) for the IGRA assays, QFT (22-76%) [43, 44], and T-SPOT.TB (77%) [44]. In a recent study, Moses *et al* estimated (using a Markov's model) that serial testing with QFT-GIT of HCWs in low-incidence areas like North America would result in a 24.6% false positive rate over a 10 year period [45]. Based on these data, the CDC currently does not recommend routine serial retesting for HCWs, except for those at increased risk for TB [46].

So far there have not been any similar data from serial retesting in patients with rheumatic diseases treated with biologics. There has been only 1 retrospective study of transplant patients who converted their QFT assay (n = 23/195, 12%) and had been retested (n = 7). Among these patients, 3/7 (43%) reverted to negative [47].

In our prospective study, 5 patients who converted and did not receive INH preventive therapy were followed for ~ 5 years. The decision not to administer INH preventive therapy was made by the treating physician based on the emerging data at the time showing a high frequency of IGRA reversion in HCWs, as discussed above [44]. None of these patients developed TB while 3 of them reverted to negative (reversion rate: 60%). Interestingly, this rate of reversion was the same for the 5 patients who had been treated with INH preventive therapy (3/5, 60%) by their physician. Despite the small number of retested patients this is a novel finding that needs to be discussed further.

Limitations of our study include its small size, the absence of serial retesting in a control group treated only with non-biologics, the setting of a low-TB-incidence area, and the low proportion of foreign-born individuals or immigrants from high-TB-burden countries. Thus, our results may not be generally applicable to patients with other co-morbidites such as HIV infection, end stage renal disease, solid organ or bone marrow or stem cell transplants, or for high-TB-incidence countries.

In conclusion, our long-term, real-life, prospective study showed a high conversion of TB screening tests (TST, IGRA) during serial retesting in a low-risk biologic-treated patient population with rheumatic diseases, which in the majority of cases was a transient phenomenon. Although the number of included patients was small, we believe that these real-life data do not support routine serial TB retesting in biologic-treated patients with rheumatic diseases, with the exception of high-risk patients or for those who present with suspicious clinical findings for TB.

## References

[1] Houben RM, Dodd PJ The Global Burden of Latent Tuberculosis Infection: A Re–estimation Using Mathematical Modelling. PLoS Med 2016;13(10):e1002152. doi:10.1371/journal.pmed.1002152. 27780211 PMC5079585

[2] Alsdurf H, Hill PC, Matteelli A, Getahun H, Menzies D The cascade of care in diagnosis and treatment of latent tuberculosis infection: a systematic review and meta–analysis. Lancet Infect Dis 2016;16(11):1269–1278. doi:10.1016/S1473-3099(16)30216-X. 27522233

[3] Lee E, Holzman RS Evolution and current use of the tuberculin test. Clin Infect Dis 2002;34(3):365–370. doi: 10.1086/338149 11774084

[4] Lobue P, Menzies D Treatment of latent tuberculosis infection: An update. Respirology 2010;15(4):603–622. doi:10.1111/j.1440-1843.2010.01751.x 20409026

[5] Wong SH, Gao Q, Tsoi KK, Wu WK, Tam LS, Lee N, Chan FK, Wu JC, Sung JJ, Ng SC Effect of immunosuppressive therapy on interferon gamma release assay for latent tuberculosis screening in patients with autoimmune diseases: a systematic review and meta–analysis. Thorax 2016;71(1):64–72. doi:10.1136/thoraxjnl-2015-207811. 26659461

[6] Lewinsohn DM, Leonard MK, LoBue PA, Cohn DL, Daley CL, Desmond E, Keane J, Lewinsohn DA, Loeffler AM, Mazurek GH, O'Brien RJ, Pai M, Richeldi L, Salfinger M, Shinnick TM, Sterling TR, Warshauer DM, Woods GL Official American Thoracic Society/Infectious Diseases Society of America/Centers for Disease Control and Prevention Clinical Practice Guidelines: Diagnosis of Tuberculosis in Adults and Children. Clin Infect Dis 2017;64(2):111–115. doi:10.1093/cid/ciw778. 28052967 PMC5504475

[7] Keane J, Gershon S, Wise RP, Mirabile-Levens E, Kasznica J, Schwieterman WD, Siegel JN, Braun MM Tuberculosis associated with infliximab, a tumor necrosis factor alpha-neutralizing agent. N Engl J Med 2001;345(15):1098–1104. doi:10.1056/NEJMoa011110. 11596589

[8] Wolfe F, Michaud K, Anderson J, Urbansky K Tuberculosis infection in patients with rheumatoid arthritis and the effect of infliximab therapy. Arthritis Rheum 2004;50(2):372–379. doi:10.1002/art.2000 14872478

[9] Solovic I, Sester M, Gomez-Reino JJ, Rieder HL, Ehlers S, Milburn HJ, Kampmann B, Hellmich B, Groves R, Schreiber S, Wallis RS, Sotgiu G, Scholvinck EH, Goletti D, Zellweger JP, Diel R, Carmona L, Bartalesi F, Ravn P, Bossink A, Duarte R, Erkens C, Clark J, Migliori GB, Lange C The risk of tuberculosis related to tumour necrosis factor antagonist therapies: a TBNET consensus statement. Eur Respir J 2010;36(5):1185–1206. doi:10.1183/09031936.00028510. 20530046

[10] Koike T, Harigai M, Inokuma S, Ishiguro N, Ryu J, Takeuchi T, Takei S, Tanaka Y, Ito K, Yamanaka H Postmarketing surveillance of tocilizumab for rheumatoid arthritis in Japan: interim analysis of 3881 patients. Ann Rheum Dis 2011;70(12):2148–2151. doi:10.1136/ard.2011.151092. 21852254 PMC3212697

[11] Winthrop KL, Park SH, Gul A, Cardiel MH, Gomez-Reino JJ, Tanaka Y, Kwok K, Lukic T, Mortensen E, Ponce de LD, Riese R, Valdez H Tuberculosis and other opportunistic infections in tofacitinib-treated patients with rheumatoid arthritis. Ann Rheum Dis 2016;75(6):1133–1138. doi:10.1136/annrheumdis-2015-207319. 26318385 PMC4893093

[12] Pettipher C, Benitha R Tuberculosis in biologic users for rheumatic diseases: results from the South African Biologics Registry (SABIO). Ann Rheum Dis 2019 [Epub ahead of print]. doi:10.1136/annrheumdis-2019-216128. 31791950

[13] Carmona L, Gomez-Reino JJ, Rodriguez-Valverde V, Montero D, Pascual-Gomez E, Mola EM, Carreno L, Figueroa M Effectiveness of recommendations to prevent reactivation of latent tuberculosis infection in patients treated with tumor necrosis factor antagonists. Arthritis Rheum 2005;52(6):1766–1772. doi:10.1002/art.21043. 15934089

[14] Singh JA, Saag KG, Bridges SL Jr., Akl EA, Bannuru RR, Sullivan MC, Vaysbrot E, McNaughton C, Osani M, Shmerling RH, Curtis JR, Furst DE, Parks D, Kavanaugh A, O'Dell J, King C, Leong A, Matteson EL, Schousboe JT, Drevlow B, Ginsberg S, Grober J, St Clair EW, Tindall E, Miller AS, McAlindon T 2015 American College of Rheumatology Guideline for the Treatment of Rheumatoid Arthritis. Arthritis Rheumatol 2016;68(1):1–26. doi:10.1002/art.39480. 26545940

[15] Kleinert S, Tony HP, Krueger K, Detert J, Mielke F, Rockwitz K, Schwenke R, Burmester GR, Diel R, Feuchtenberger M, Kneitz C Screening for latent tuberculosis infection: performance of tuberculin skin test and interferon-gamma release assays under real–life conditions. Ann Rheum Dis 2012;71(11):1791–1795. doi:10.1136/annrheumdis-2011-200941. 22586160

[16] Winthrop KL, Weinblatt ME, Daley CL You can't always get what you want, but if you try sometimes (with two tests–TST and IGRA–for tuberculosis) you get what you need. Ann Rheum Dis 2012;71(11):1757–1760. doi:10.1136/annrheumdis-2012-201979. 22975752

[17] Vassilopoulos D, Tsikrika S, Hatzara C, Podia V, Kandili A, Stamoulis N, Hadziyannis E Comparison of two gamma interferon release assays and tuberculin skin testing for tuberculosis screening in a cohort of patients with rheumatic diseases starting anti-tumor necrosis factor therapy. Clin Vaccine Immunol 2011;18(12):2102–2108. doi:10.1128/CVI.05299-11. 21994356 PMC3232699

[18] Gran G, Assmus J, Dyrhol-Riise AM Screening for latent tuberculosis in Norwegian health care workers: high frequency of discordant tuberculin skin test positive and interferon–gamma release assay negative results. BMC Public Health 2013;13(353. doi:10.1186/1471-2458-13-353. PMC3637593 23590619

[19] Pai M, Banaei N Occupational screening of health care workers for tuberculosis infection: tuberculin skin testing or interferon-gamma release assays? Occup Med (Lond) 2013;63(7):458–460. doi:10.1093/occmed/kqt105. 24097956

[20] Hatzara C, Hadziyannis E, Kandili A, Koutsianas C, Makris A, Georgiopoulos G, Vassilopoulos D Frequent conversion of tuberculosis screening tests during anti-tumour necrosis factor therapy in patients with rheumatic diseases. Ann Rheum Dis 2015;74(10):1848–1853. doi:10.1136/annrheumdis-2014-205376. 24854354

[21] Kim KH, Lee SW, Chung WT, Kim BG, Woo KS, Han JY, Kim JM Serial interferon-gamma release assays for the diagnosis of latent tuberculosis infection in patients treated with immunosuppressive agents. Korean J Lab Med 2011;31(4):271–278. doi:10.3343/kjlm.2011.31.4.271. 22016681 PMC3190006

[22] Jung YJ, Woo HI, Jeon K, Koh WJ, Jang DK, Cha HS, Koh EM, Lee NY, Kang ES The Significance of Sensitive Interferon Gamma Release Assays for Diagnosis of Latent Tuberculosis Infection in Patients Receiving Tumor Necrosis Factor-alpha Antagonist Therapy. PLoS One 2015;10(10):e0141033. doi:10.1371/journal.pone.0141033. 26474294 PMC4608840

[23] Son CN, Jun JB, Kim JH, Sung IH, Yoo DH, Kim TH Follow-up testing of inter-feron-gamma release assays are useful in ankylosing spondylitis patients receiving anti-tumor necrosis factor alpha for latent tuberculosis infection. J Korean Med Sci 2014;29(8):1090–1093. doi:10.3346/jkms.2014.29.8.1090. 25120318 PMC4129200

[24] WHO. Global tuberculosis control 2019. Accessed at: https://www.who.int/tb/publications/global_report/en/ on 12 29, 2019.

[25] Hsia EC, Cush JJ, Matteson EL, Beutler A, Doyle MK, Hsu B, Xu S, Rahman MU Comprehensive tuberculosis screening program in patients with inflammatory arthri-tides treated with golimumab, a human anti-tumor necrosis factor antibody, in Phase III clinical trials. Arthritis Care Res (Hoboken) 2013;65(2):309–313. doi:10.1002/acr.21788. 22782640

[26] Chen DY, Shen GH, Chen YM, Chen HH, Hsieh CW, Lan JL Biphasic emergence of active tuberculosis in rheumatoid arthritis patients receiving TNFalpha inhibitors: the utility of IFNgamma assay. Ann Rheum Dis 2012;71(2):231–237. doi:10.1136/annrheumdis-2011-200489. 22021896

[27] Iannone F, Cantini F, Lapadula G Diagnosis of latent tuberculosis and prevention of reactivation in rheumatic patients receiving biologic therapy: international recommendations. J Rheumatol 2014;Suppl. 91:41–46. doi:10.3899/jrheum.140101. 24788999

[28] Menter A, Strober BE, Kaplan DH, Kivelevitch D, Prater EF, Stoff B, Armstrong AW, Connor C, Cordoro KM, Davis DMR, Elewski BE, Gelfand JM, Gordon KB, Gottlieb AB, Kavanaugh A, Kiselica M, Korman NJ, Kroshinsky D, Lebwohl M, Leonardi CL, Lichten J, Lim HW, Mehta NN, Paller AS, Parra SL, Pathy AL, Rupani RN, Siegel M, Wong EB, Wu JJ, Hariharan V, Elmets CA Joint AAD-NPF guidelines of care for the management and treatment of psoriasis with biologics. J Am Acad Dermatol 2019;80(4):1029–1072. doi:10.1016/j.jaad.2018.11.057. 30772098

[29] Tran NQ, Garcia-Rosell M, Pattanaik D, Raza SH, Carbone L Screening and Treatment of Latent Tuberculosis Among Patients Receiving Biologic Agents: A National and International Survey of Rheumatologists. J Clin Rheumatol 2017;23(1):6–11. doi:10.1097/RHU.0000000000000466. 28002150

[30] Chen DY, Shen GH, Hsieh TY, Hsieh CW, Lan JL Effectiveness of the combination of a whole-blood interferon-gamma assay and the tuberculin skin test in detecting latent tuberculosis infection in rheumatoid arthritis patients receiving adalimumab therapy. Arthritis Rheum 2008;59(6):800–806. doi:10.1002/art.23705. 18512714

[31] Park JH, Seo GY, Lee JS, Kim TH, Yoo DH Positive conversion of tuberculin skin test and performance of interferon release assay to detect hidden tuberculosis infection during anti-tumor necrosis factor agent trial. J Rheumatol 2009;36(10):2158–2163. doi:10.3899/jrheum.090150. 19723901

[32] Garcovich S, Ruggeri A, D'Agostino M, Ardito F, De SC, Delogu G, Fadda G Clinical applicability of Quantiferon-TB-Gold testing in psoriasis patients during long-term anti-TNF-alpha treatment: a prospective, observational study. J Eur Acad Dermatol Venereol 2012;26(12):1572–1576. doi:10.1111/j.1468-3083.2011.04220.x. 21923840

[33] Hatemi G, Melikoglu M, Ozbakir F, Tascilar K, Yazici H Quantiferon-TB Gold in tube assay for the screening of tuberculosis before and during treatment with tumor necrosis factor alpha antagonists. Arthritis Res Ther 2012;14(3):R147. doi:10.1186/ar3882. 22709461 PMC3446532

[34] Papay P, Primas C, Eser A, Novacek G, Winkler S, Frantal S, Angelberger S, Mikulits A, Dejaco C, Kazemi-Shirazi L, Vogelsang H, Reinisch W Retesting for latent tuberculosis in patients with inflammatory bowel disease treated with TNF-alpha inhibitors. Aliment Pharmacol Ther 2012;36(9):858–865. doi: 10.1111/apt.12037. 22978645

[35] Scrivo R, Sauzullo I, Mengoni F, Priori R, Coppola M, Iaiani G, DI FM, Vullo V, Mastroianni CM, Valesini G Mycobacterial interferon-gamma release variations during longterm treatment with tumor necrosis factor blockers: lack of correlation with clinical outcome. J Rheumatol 2013;40(2):157–165. doi:10.3899/jrheum.120688. 23204217

[36] Abreu C, Afonso J, Camila DC, Ruas R, Sarmento A, Magro F Serial Tuberculosis Screening in Inflammatory Bowel Disease Patients Receiving Anti-TNFalpha Therapy. J Crohns Colitis 2017;11(10):1223–1229. doi:10.1093/ecco-jcc/jjx080. 28605520

[37] Lee CK, Wong SHV, Lui G, Tang W, Tam LS, Ip M, Hung E, Chen M, Wu JC, Ng SC A Prospective Study to Monitor for Tuberculosis During Anti-tumour Necrosis Factor Therapy in Patients With Inflammatory Bowel Disease and Immune-mediated Inflammatory Diseases. J Crohns Colitis 2018;12(8):954–962. doi:10.1093/ecco-jcc/jjy057. 29757355

[38] Taxonera C, Ponferrada A, Riestra S, Bermejo F, Saro C, Martin-Arranz MD, Cabriada JL, Barreiro-de AM, de Castro ML, Lopez-Serrano P, Barrio J, Suarez C, Iglesias E, Arguelles-Arias F, Ferrer I, Marin-Jimenez I, Hernandez-Camba A, Bastida G, Van DM, Martinez-Montiel P, Olivares D, Rivero M, Fernandez-Salazar L, Nantes O, Merino O, Alba C, Gisbert JP Serial Tuberculin Skin Tests Improve the Detection of Latent Tuberculosis Infection in Patients With Inflammatory Bowel Disease. J Crohns Colitis 2018;12(11):1270–1279. doi:10.1093/ecco-jcc/jjy104. 30052856

[39] He D, Bai F, Zhang S, Jiang T, Shen J, Zhu Q, Yue T, Shao L, Gao Y, Feng Y, Weng X, Zou H, Zhang Y, Zhang W High incidence of tuberculosis infection in rheumatic diseases and impact for chemoprophylactic prevention of tuberculosis activation during biologics therapy. Clin Vaccine Immunol 2013;20(6):842–847. doi:10.1128/CVI.00049-13. 23554465 PMC3675964

[40] Chung J, Aronson AB, Srikantha R, Vogelgesang SA, Wanat KA Low conversion rate of QuantiFERON-TB Gold screening tests in patients treated with tumor necrosis factor inhibitors: A retrospective cohort study identifying an important practice gap. J Am Acad Dermatol 2018;79(1):169–171. doi:10.1016/j.jaad.2018.03.025. 29588247

[41] Kim HW, Kwon OC, Han SH, Park MC Positive conversion of interferon-gamma release assay in patients with rheumatic diseases treated with biologics. Rheumatol Int 2020 [Epub ahead of print]. doi: 10.1007/s00296-019-04510-6. 31919575

[42] Scrivo R, Sauzullo I, Mengoni F, Iaiani G, Vestri AR, Priori R, Di FE, Di FM, Spinelli FR, Vullo V, Mastroianni CM, Valesini G Serial interferon-gamma release assays for screening and monitoring of tuberculosis infection during treatment with biologic agents. Clin Rheumatol 2012;31(11):1567–1575. doi:10.1007/s10067-012-2049-6 22864811

[43] Nienhaus A, Ringshausen FC, Costa JT, Schablon A, Tripodi D IFN-gamma release assay versus tuberculin skin test for monitoring TB infection in healthcare workers. Expert Rev Anti Infect Ther 2013;11(1):37–48. doi:10.1586/eri.12.150. 23428101

[44] Dorman SE, Belknap R, Graviss EA, Reves R, Schluger N, Weinfurter P, Wang Y, Cronin W, Hirsch-Moverman Y, Teeter LD, Parker M, Garrett DO, Daley CL Interferon-gamma Release Assays and Tuberculin Skin Testing for Diagnosis of Latent Tuberculosis Infection in Healthcare Workers in the United States. Am J Respir Crit Care Med 2014;189(1):77–87. doi:10.1164/rccm.201302-0365OC. 24299555

[45] Moses MW, Zwerling A, Cattamanchi A, Denkinger CM, Banaei N, Kik SV, Metcalfe J, Pai M, Dowdy D Serial testing for latent tuberculosis using QuantiFERON-TB Gold In-Tube: A Markov model. Sci Rep 2016;6:30781. doi:10.1038/srep30781. 27469388 PMC4965809

[46] Sosa LE, Njie GJ, Lobato MN, Bamrah MS, Buchta W, Casey ML, Goswami ND, Gruden M, Hurst BJ, Khan AR, Kuhar DT, Lewinsohn DM, Mathew TA, Mazurek GH, Reves R, Paulos L, Thanassi W, Will L, Belknap R Tuberculosis Screening, Testing, and Treatment of U.S. Health Care Personnel: Recommendations from the National Tuberculosis Controllers Association and CDC, 2019. MMWR Morb Mortal Wkly Rep 2019;68(19):439–443. doi:10.15585/mmwr.mm6819a3.51 31099768 PMC6522077

[47] Roth PJ, Grim SA, Gallitano S, Adams W, Clark NM, Layden JE Serial testing for latent tuberculosis infection in transplant candidates: a retrospective review. Transpl Infect Dis 2016;18(1):14–21. doi:10.1111/tid.12489. 26671024

